# Glucose Metabolism, Islet Architecture, and Genetic Homogeneity in Imprinting of [Ca^2+^]_i_ and Insulin Rhythms in Mouse Islets

**DOI:** 10.1371/journal.pone.0008428

**Published:** 2009-12-23

**Authors:** Craig S. Nunemaker, John F. Dishinger, Stacey B. Dula, Runpei Wu, Matthew J. Merrins, Kendra R. Reid, Arthur Sherman, Robert T. Kennedy, Leslie S. Satin

**Affiliations:** 1 Department of Medicine, Division of Endocrinology and Metabolism, University of Virginia, Charlottesville, Virginia, United States of America; 2 Department of Chemistry, University of Michigan, Ann Arbor, Michigan, United States of America; 3 Department of Pharmacology, University of Michigan, Ann Arbor, Michigan, United States of America; 4 Brehm Diabetes Center, University of Michigan, Ann Arbor, Michigan, United States of America; 5 Laboratory of Biological Modeling, National Institutes of Health, Bethesda, Maryland, United States of America; University of Bremen, Germany

## Abstract

We reported previously that islets isolated from individual, outbred Swiss-Webster mice displayed oscillations in intracellular calcium ([Ca^2+^]_i_) that varied little between islets of a single mouse but considerably between mice, a phenomenon we termed “islet imprinting.” We have now confirmed and extended these findings in several respects. First, imprinting occurs in both inbred (C57BL/6J) as well as outbred mouse strains (Swiss-Webster; CD1). Second, imprinting was observed in NAD(P)H oscillations, indicating a metabolic component. Further, short-term exposure to a glucose-free solution, which transiently silenced [Ca^2+^]_i_ oscillations, reset the oscillatory patterns to a higher frequency. This suggests a key role for glucose metabolism in maintaining imprinting, as transiently suppressing the oscillations with diazoxide, a K_ATP_-channel opener that blocks [Ca^2+^]_i_ influx downstream of glucose metabolism, did not change the imprinted patterns. Third, imprinting was not as readily observed at the level of single beta cells, as the [Ca^2+^]_i_ oscillations of single cells isolated from imprinted islets exhibited highly variable, and typically slower [Ca^2+^]_i_ oscillations. Lastly, to test whether the imprinted [Ca^2+^]_i_ patterns were of functional significance, a novel microchip platform was used to monitor insulin release from multiple islets in real time. Insulin release patterns correlated closely with [Ca^2+^]_i_ oscillations and showed significant mouse-to-mouse differences, indicating imprinting. These results indicate that islet imprinting is a general feature of islets and is likely to be of physiological significance. While islet imprinting did not depend on the genetic background of the mice, glucose metabolism and intact islet architecture may be important for the imprinting phenomenon.

## Introduction

Although many genetic and environmental factors contribute to the development of type 2 diabetes, one of the key components is the failure of the pancreatic beta-cell to secrete insulin appropriately in the face of insulin resistance. In healthy individuals, beta-cells respond to glucose in a well-defined manner. As blood glucose levels rise, glucose is taken into the beta cell through glucose transporters and is metabolized through glycolysis and aerobic respiration, leading to an increase in ATP/ADP. An increase in ATP/ADP results in the closure of ATP-sensitive potassium channels (K_ATP_), which triggers calcium influx through voltage-gated Ca^2+^ channels and results in insulin release. Following an initial burst of calcium influx and insulin secretion, beta-cells within the islets typically generate oscillations in intracellular calcium ([Ca^2+^]_i_) and insulin release [1]–[6] that continue as long as glucose remains elevated. These features of beta-cell function occur both *in vitro* and *in vivo* [7], [8].

The islet behaves as a functional syncytium because its constituent beta cells are electrically coupled to one another via gap junctions [9]–[12]. Additional endocrine cell types in the islets, chiefly the glucagon-secreting alpha-cells, also influence islet function. When islets are dispersed into individual beta cells and tissue cultured, they retain the capacity to generate [Ca^2+^]_i_ oscillations in response to glucose, but the dynamics of these oscillations and their sensitivity to glucose differ from those of intact islets [13]–[15]. Intact islet architecture is thus an important influence on beta-cell stimulus-secretion coupling.

Islet [Ca^2+^]_i_ oscillations and the pulses of insulin secretion they drive are modulated by a large number of extrinsic factors, including glucose [8], amino acids [16], fatty acids [17], and neurotransmitters [18], [19]. We reported for the first time that an additional factor is the individual mouse selected as a source for the islets [20]. Islet [Ca^2+^]_i_ oscillations recorded from a population of different mice are heterogeneous and can be broadly classified into “fast” (period <2 minutes) or “slow” (period >2 minutes), with some slow patterns exhibiting faster oscillations superimposed upon the slower oscillations (“mixed”, classified as slow by their period); yet islets isolated from *individual* Swiss-Webster mice had glucose-dependent [Ca^2+^]_i_ oscillations that appeared to be tightly distributed in terms of period [20]. All of the mice studied were of the same sex and were close in age and in body weight. We also ruled out potential sources of conformity including the islet isolation and culture methods used. Importantly, while we did not record insulin secretion *in vitro* from the islets being studied, relying instead on [Ca^2+^]_i_ oscillations as a surrogate for insulin secretion, insulin pulsatility was monitored in the same mice *in vivo* with a hyperglycemic glucose clamp. This allowed us to later correlate *in vitro* islet [Ca^2+^]_i_ oscillations with *in vivo* insulin pulsatility in groups of islets taken from these same individual mice. When plotted, these data yielded a linear relationship between the periods of the insulin pulses representative of a given mouse and the islet [Ca^2+^]_i_ periods seen *ex vivo*. The results were interpreted as evidence that oscillations in islet [Ca^2+^]_i_ are an important driver of *in vivo* plasma insulin oscillations [20].

However, a number of major questions remained: 1) Is imprinting peculiar to the outbred Swiss-Webster strain we studied, or is it a more general phenomenon? Closely related to this is whether imprinting can be observed in inbred mice, which might be expected to be less variable. 2) Is imprinting dependent on non-genetic factors such as glucose metabolism? 3) Does imprinting extend to the isolated beta cell or is intact islet architecture required? And lastly, 4) What is the significance of imprinted islet [Ca^2+^]_i_ oscillations for oscillations of insulin secretion from those same islets? Addressing these key questions is the focus of this report. Our results suggest that imprinting is a more general phenomenon of mouse models that applies to oscillations in insulin secretion as well as [Ca^2+^]_i_. Further, the pattern exhibited by islets depends on their history of exposure to glucose in addition to the instantaneous glucose concentration, and imprinting requires intact islet architecture for it to be clearly manifested. The implications of imprinting and its possible mechanisms are discussed.

## Materials and Methods

### Mice and Islet Isolation

Male mice from CD1, Swiss-Webster, and C57Bl/6J strains weighing 20–35 grams (unless otherwise stated) were housed in a pathogen-free facility at the University of Virginia (UVA), Virginia Commonwealth University (VCU), or the University of Michigan. Mice were euthanized according to IACUC approved protocols at each institution, and pancreatic islets were isolated by collagenase digestion and purified by hand-picking [20] or Histopaque centrifugation [21], as previously described. Following isolation, islets were transferred to a petri dish containing RPMI 1640 supplemented with 11 mM glucose, 10% (v/v) fetal bovine serum, 100 U/mL penicillin, and 100 µg/mL streptomycin. (Invitrogen, Carlsbad, CA). All islets were incubated overnight in culture to allow sufficient recovery time from collagenase digestion before any experiments were performed.

### Beta-Cell Preparation

Islets were transferred to a Sigmacoted test tube containing 5 ml of calcium-free Spinner salt solution containing in g/L: 6.8 NaCl, 0.4 KCl, 0.2 MgSO_4_, 2.2 NaHCO_3_, 1.4 NaH_2_CO_4_, 1.0 glucose, 0.01 phenol red, plus on the day of use 1.14 g EGTA and 1.0 g BSA; pH 7.4) and gently triturated against the side of the tube using a Sigmacoted pipette. Islets were then incubated in Spinner salt solution for 8–10 minutes at 37°C and twice centrifuged at 800 rpm for 5 minutes and washed with KRB solution. After centrifuging a third time, cells were resuspended in RPMI-1640 media and plated onto glass cover slips coated with 0.1% gelatin. Since rodent islets are comprised of ∼80% beta cells, ∼10–15% alpha cells, and ∼5% other cell types [22], we reduced the possibility of recording non-beta cells by subjecting all cells to 3 mM glucose following the initial recording of [Ca^2+^]_i_ oscillations in 11 mM glucose. Cells that did not become inactive in 3 mM glucose were considered possible non-beta cells (alpha or delta) and were excluded from the study [23]–[25] leaving a <∼5% likelihood of including a non-beta cell in the data set.

### Microchip Fabrication and Operation

**Figure 1:**
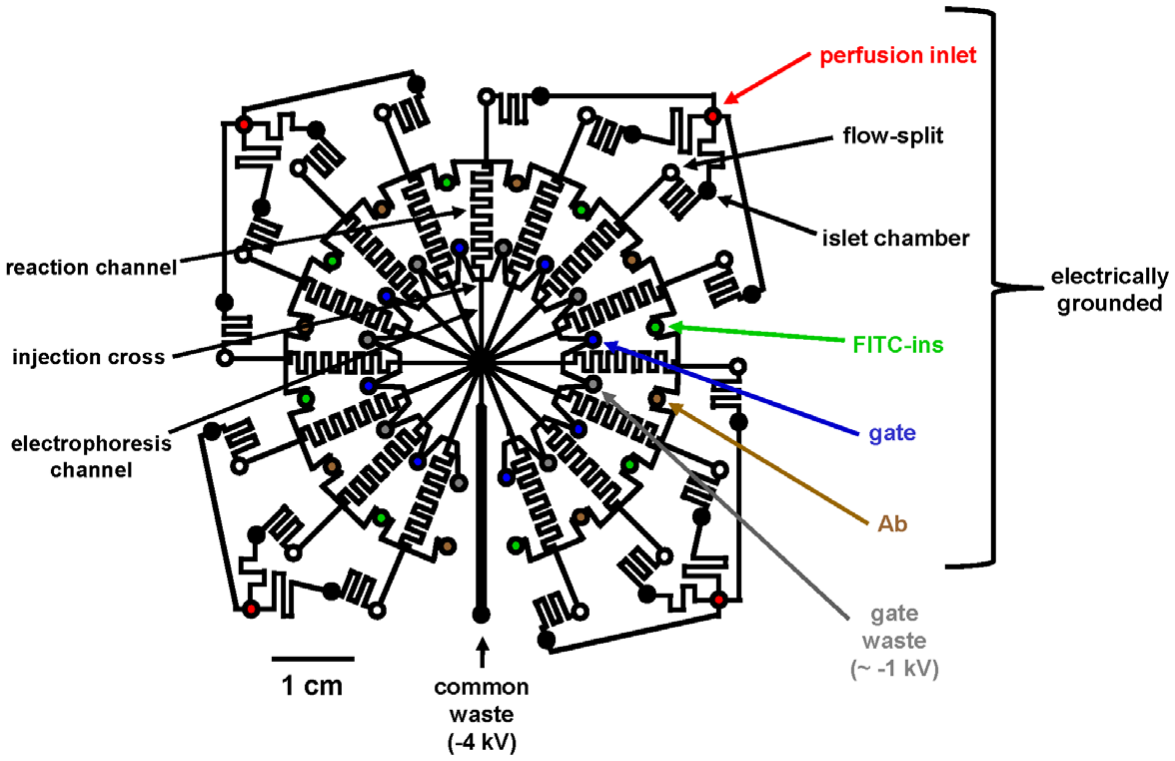
Channel layout of a microfluidic chip for measuring insulin secretion. Solid black lines indicate microfluidic channels and fluid reservoirs (circles) are color coded for clarity. The chip is capable of measuring insulin secretion in real time from 15 independent islets simultaneously. Refer to Materials and Methods and [26] for additional details.

Insulin secretion was recorded from up to 15 individual islets at a time in parallel using a microfluidic chip fabricated as described previously [26]. The chip, illustrated in Figure 1, consists of 15 channel networks (channels 15 µm deep, approximately 35 µm wide) each capable of automated single islet analysis with capillary electrophoresis-based immunoassays [27]. Briefly, islets loaded into individual chambers were maintained at 37°C and perfused with a balanced salt solution containing (in mM): 125 NaCl, 5.9 KCl, 1.2 MgCl_2_, 2.4 CaCl_2_, 25 tricine, 0.7 mg/mL albumin, pH 7.4 at 500 nL/min. This flow rate washes out the single islet chambers out every 3.5 s.

Flow coming from the islet chamber carrying secreted insulin was split so that most went to waste while a small portion entered a microfluidic cross where it was mixed on-chip with FITC-labeled insulin (FITC-ins) and anti-insulin antibody (Ab). FITC-ins and Ab were pumped from their respective reservoirs to the serpentine reaction channel by electroosmotic flow (EOF). The resulting reaction stream was periodically injected onto the electrophoresis channel by manipulating the EOF at the injection cross, as previously described [26]. The resulting FITC-insulin and FITC-insulin:Ab complexes were separated by electrophoresis in the channels. Detection of the separated zones was accomplished by imaging the center region of the chip with a fluorescence microscope (IX71 microscope, Olympus; LB-LS/30 Xe arc lamp, Sutter Instrument Co.; FITC filter cube, Semrock; C9100-13 EMCCD camera, Hamamatsu Photonic Systems) where all electrophoresis channels converged. Images were collected at 28 Hz using a 20x objective (Olympus) and analyzed using SlideBook software (Intelligent Imaging Innovations, Inc.). Images were processed to produce electropherograms correlating to each channel network with 10 s temporal resolution for each islet. The resulting traces were converted to insulin secretion by calibration curves. This system allows insulin to be monitored from all islets individually at 10 s intervals.

### Intracellular Calcium ([Ca^2+^]_i_)

[Ca^2+^]_i_ was measured using the ratiometric indicator fura-2 AM using methods that have been previously described [28]. Briefly, islets were loaded with 3 uM fura-2 AM (30–40 min), washed, and then transferred to a small volume chamber (Warner Instruments, Hamden, CT) mounted on the stage of an Olympus BX51WI fluorescence microscope (Olympus, Tokyo, Japan). Beta cells were transferred on glass shards to the recording chamber following fura-2 loading as described above. Islets or beta cells were perifused and imaged as described in [28]. For [Ca^2+^]_i_ measurements related to the microfluidics studies of insulin secretion, fura-2 images were recorded using different equipment described in (28). A subset of data included in this manuscript was recorded using the [Ca^2+^]_i_ indicator fluo-3 on an Olympus BX61W1 upright laser-scanning confocal microscope using the FluoView acquisition system (excitation/emission of 488/535 nm; Olympus, Tokyo, Japan) as described previously [6].

### Measurements of Endogenous NAD(P)H Fluorescence in Islets

NAD(P)H was measured in islets by the autofluorescence of the naturally occurring pyridine nucleotides NADH and NADPH. Islet NAD(P)H fluorescence [5], [29] was elicited by 365 nm light from a Till Polychrome V (Till Photonics, Graefling, Germany), and filtered via a 410dclp beamsplitter and a D500/100 wide-band emission filter (Chroma Technology, Rockingham, VT) mounted on an Olympus IX-71 inverted microscope. Emission intensity was collected by a Photometrics Quant-EM camera at 5-sec intervals. The recording solution was maintained at 30–33°C by resistive elements in both an inline heater and in the RC-26 recording chamber itself (Warner Instruments, Hamden, CT).

### Data Analysis and Statistics

The periods of [Ca^2+^]_i_ and insulin oscillations were measured using CLUSTER8 and direct measurement as previously described [28]. NAD(P)H oscillations were analyzed using Welch fast Fourier transform with Hanning smoothing using Matlab (MathWorks, Natick, MA). A two-tailed t-test was used for comparisons between beta cells and islets and a one-way ANOVA was used to compare oscillatory patterns among mice followed by a Tukey post-test. A p-value of p<0.05 was used as an indication of statistical significance. Statistical analysis was performed using Prism and In-Stat software (Graphpad Software, Inc., La Jolla, CA).

## Results

### Both Inbred and Outbred Mouse Strains Exhibit Imprinting

**Figure 2:**
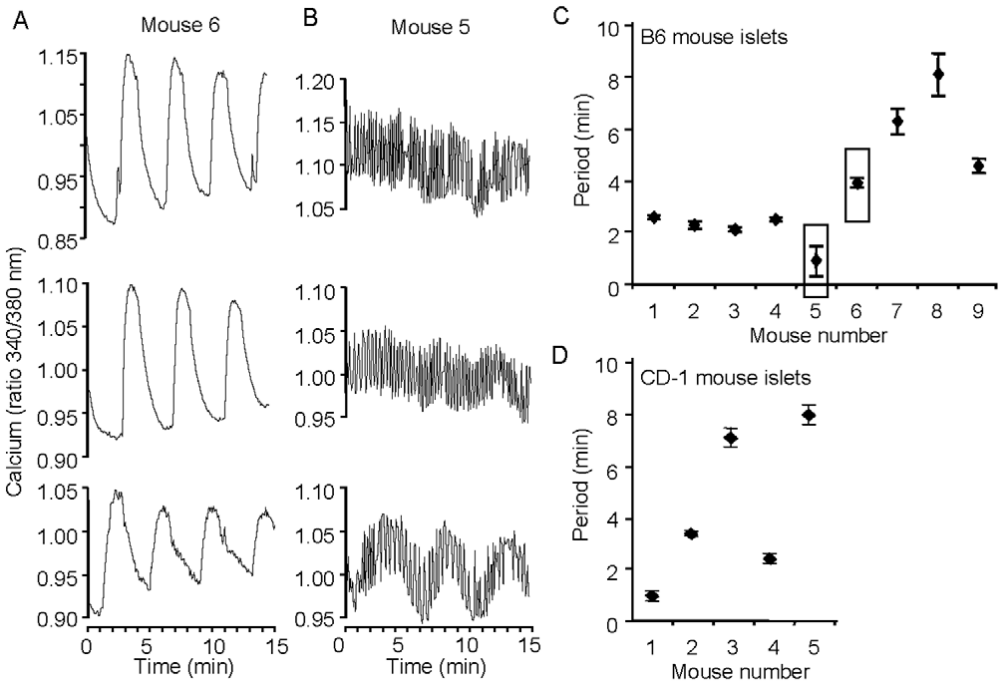
Both inbred and outbred mice display islet imprinting. (A–B) Two representative C57BL/6J mice out of a group of 9 displayed very different [Ca^2+^]_i_ oscillation patterns. Three representative islets from Mouse 6 display slow oscillations (A, period: 3.9±0.2 minutes, n = 12 islets total) and three representative islets from Mouse 5 display fast oscillations (B, period: 0.9±0.6 minutes, n = 9 islets total). One trace shown in B (bottom) shows a clear ‘slow component’ that was representative of n = 4 islets from Mouse 5 (period: 5.4±0.1 minutes). (C–D) The variation in the period of [Ca^2+^]_i_ oscillations indicates distinct differences from mouse to mouse for the inbred B6 strain (C) and the outbred CD-1 strain (D), as shown by one-way ANOVA (p<1.0e-24). Boxes drawn around Mouse 6 and Mouse 5 in (C) are described above.

The imprint phenomenon was first reported in Swiss-Webster mice, an outbred strain [20]. This naturally leads to the possibility that imprinting might reflect heterogeneity in the genetic backgrounds from one mouse to the next. To determine if imprinting also occurs among mice from an identical genetic background, we examined C57Bl/6J mice, a commonly used inbred control strain. Note that each figure in the following sections is labeled numerically in a similar manner (mouse 1, mouse 2, etc.), but each figure represents a unique set of mice. As shown in Figure 2, the [Ca^2+^]_i_ oscillations from 3 representative islets isolated from a given mouse (Mouse 6) exhibited extremely similar [Ca^2+^]_i_ oscillation patterns in 11 mM glucose under steady state conditions (Figure 2A). Similarly, islets from a different mouse (Mouse 5) also displayed uniform [Ca^2+^]_i_ patterns (Figure 2B) but had a greatly reduced oscillatory period compared to Mouse 6. A subset of islets from Mouse 5 also showed a slow rhythmic component, as can be seen in the bottom trace of Figure 2B. This type of mixed pattern has been reported previously in islet electrical activity [30], [31], intracellular calcium [8], [20], [32], and in oxygen tension [33]. We have proposed that the mixed pattern is caused by a combination of glycolytic and ionic mechanisms [8]. Using a mathematical model that incorporates both glycolytic and ionic mechanism, we have demonstrated with simulations that small quantitative variation of parameters can produce mixed patterns in which fast oscillations appear either only on the peaks of the slow oscillations or in both the peaks and troughs (compare Figures 5 and 6 in [34]). Because the amplitude of the slow component was small in Mouse 5 and could only be detected by our classification algorithm when the fast component was removed by smoothing, we report only the fast frequency in Figure 2C.

**Figure 3:**
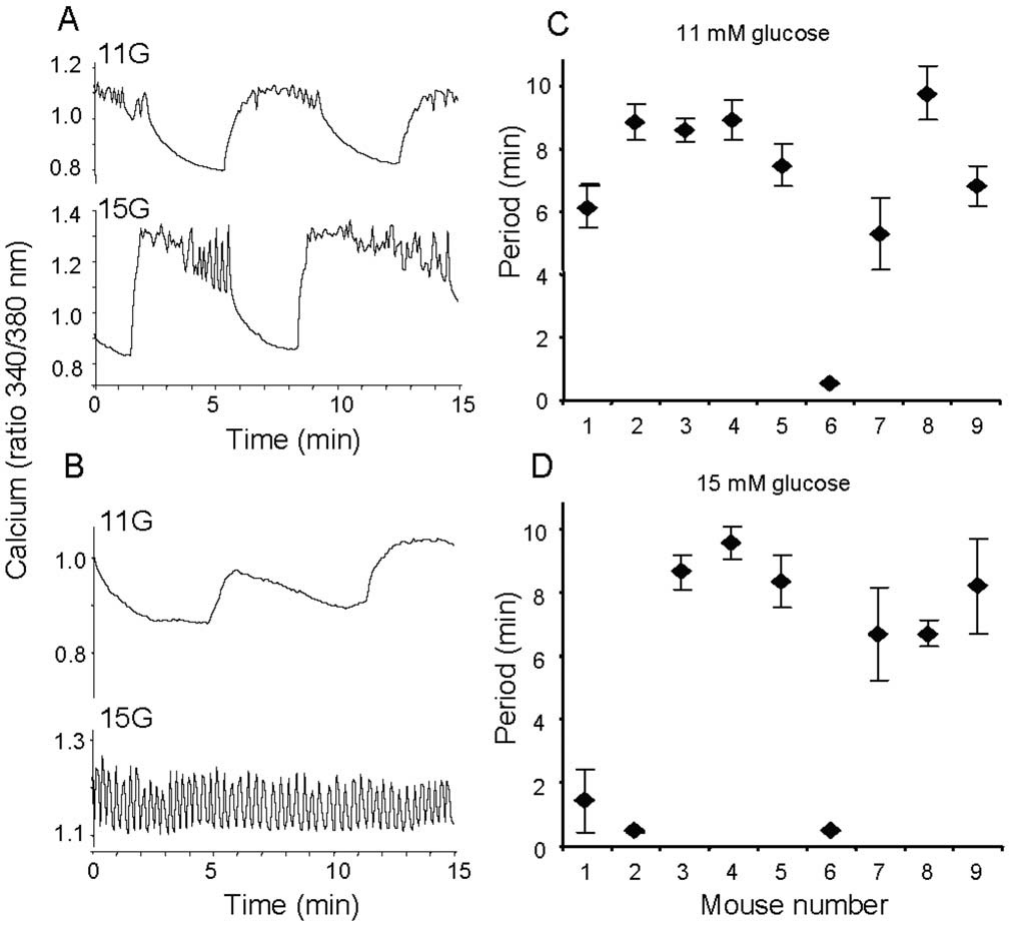
Imprinting at different glucose concentrations. (A–B) Examples of [Ca^2+^]_i_ patterns in 11 mM glucose (top) and 15 mM glucose (bottom). Patterns shown in (A) were representative of islets from 5 mice, in which the oscillations were prolonged at the higher glucose concentration. Patterns shown in (B) were representative of islets from 2 mice in which patterns switched from slow to very fast oscillations. (C–D) Mean period of oscillations among islets (n>6 islets per mouse) from 9 CD-1 mice in 11 mM glucose (C) or 15 mM glucose (D). Mouse numbers correspond to both C and D.

**Figure 4:**
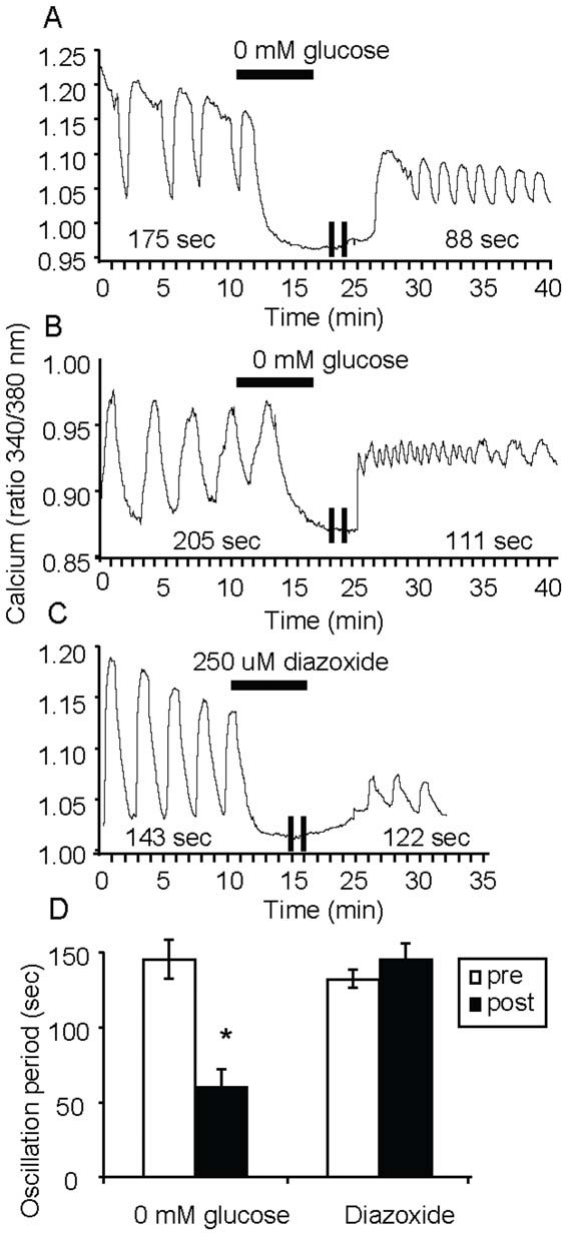
Transient reductions in glucose disrupt islet [Ca^2+^]_i_ oscillations. (A–C) Examples of islets incubated in 11 mM glucose for 10 minutes. Glucose-free saline (A,B) or saline with 11 mM glucose plus 250 uM diazoxide (C) was applied to islets for 15 minutes and then saline with 11 mM glucose was reintroduced. [Ca^2+^]_i_ oscillations following these maneuvers differ sharply from the pretreatment shown in A and B, but not for C, as indicated by the period (given in seconds) in each panel. Note that the two vertical lines shown indicate a 5-min pause in the recording during treatment. (D) Mean period of [Ca^2+^]_i_ oscillations before (pre) or after (post) either 0 mM glucose (n = 39 islets) or 250 uM diazoxide (n = 46 islets).

As summarized in Figure 2C, the periods of the [Ca^2+^]_i_ oscillations observable within a group of mice varied substantially among individual mice, whereas the periods of the [Ca^2+^]_i_ oscillations seen in islets from the same mouse were very tightly distributed. We also examined an additional outbred strain, the CD1 mouse, with similar results shown in Figure 2D. These findings indicate that the imprinting phenomenon is common to multiple strains of mice.

### Imprinting at Different Glucose Concentrations

Because islet oscillations vary with changes in glucose, we compared oscillations at glucose concentrations of 11 and 15 mM in islets from the same mouse to determine whether imprinting would persist. Islets that were recorded in 15 mM glucose were exposed to 15 mM glucose ∼45 min prior to the start of the recording; islets recorded in 11 mM glucose were maintained in 11 mM glucose prior to the experiment. As shown in Figure 3, islets from 9 different CD-1 mice produced oscillations in both 11 and 15 mM glucose. The period of these oscillation differed from mouse to mouse, but the oscillatory periods were quite similar among islets from the same mouse, which indicates imprinting. For a majority of mice, the period of islet oscillations was slightly greater in 15 mM glucose compared to 11 mM, as represented in Figure 3A. For 2 mice, the islets were much faster in 15 mM than 11 mM glucose, as represented in Figure 3B. The mean oscillation periods among islets for each mouse are shown for 11 mM glucose in Figure 3C and for 15 mM in Figure 3D. For Mouse 6, all islets produced fast oscillations in both 11 and 15 mM glucose, and for Mouse 8, oscillations were slightly faster in 15 mM glucose compared to 11 mM. Collectively, these observations are consistent with previous reports of islet oscillations at different glucose concentrations [8], [35] and with our model describing glycolytic and ionic mechanisms of producing oscillations [36].

**Figure 5:**
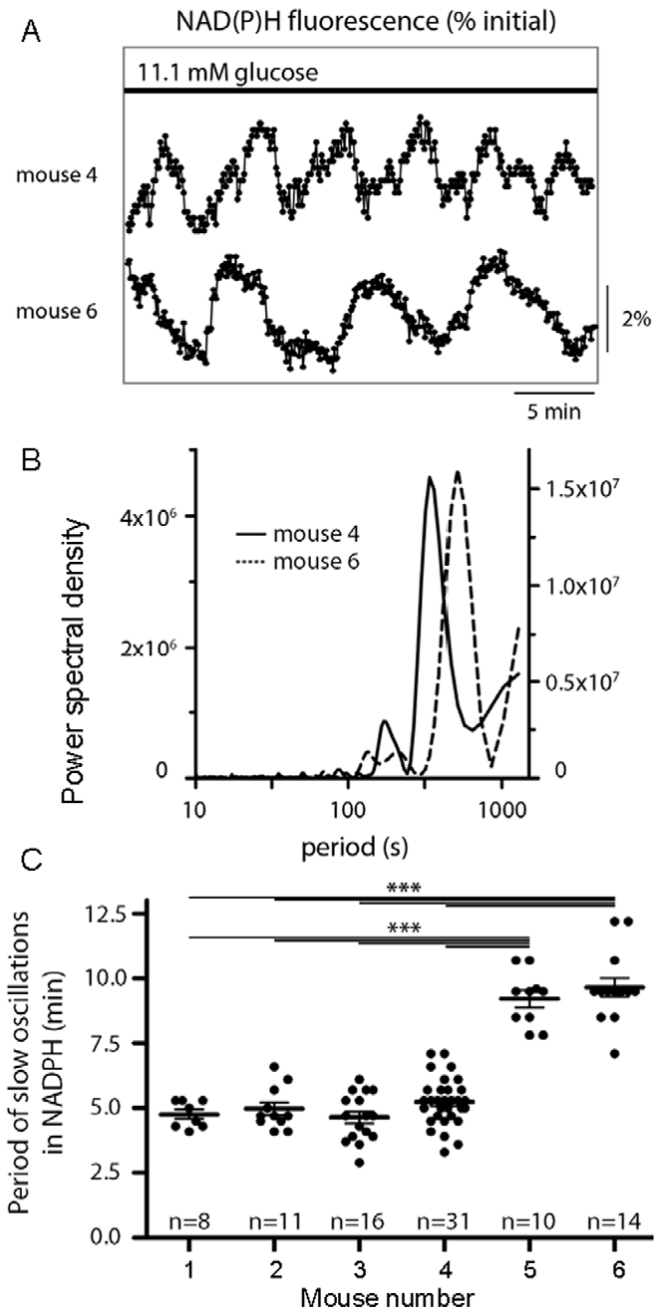
Imprinting of NAD(P)H oscillations in islets. (A) Representative examples of NAD(P)H oscillations recorded from islets from two different mice. (B) Peaks in FFT spectral power density correspond with oscillatory frequency (inverse of period) for the two examples in (A). (C) Mean period and distribution of oscillations among 6 different Swiss-Webster mice. The period of islet NAD(P)H oscillations differed among mice as measured by one-way ANOVA (p<0.0001). The number of islets is listed for each mouse in (C).

### Transient Reductions in Glucose Disrupt Islet [Ca^2+^]_i_ Oscillations

We have previously reported that transient removal of glucose concentrations can result in a switch from slow to fast oscillations when glucose concentration is restored. In contrast to the maneuvers in the previous paragraph, this resetting can change frequency at a given glucose concentration. Having shown that imprinting is preserved in the former case, we wanted to determine whether it is preserved after transient removal of glucose. After recording islet oscillations in constant 11 mM glucose for 10 min, we exposed islets to glucose-free saline for 15 min and then returned the islets to 11 mM glucose. As shown in Figure 4, the period of the islet oscillations remained constant while the islets were continually exposed to 11 mM glucose for the first 10 minutes. The application of glucose-free saline, however, caused the islets to transiently repolarize and cease oscillating until elevated glucose was restored. As shown by the representative examples in Figure 4A–B, once saline containing 11 mM glucose was reapplied to the islets, they either immediately reestablished a regular oscillatory pattern having a higher frequency (Figure 4A), or the islets exhibited a variable pattern beginning with a very fast pattern (<30 sec period) followed by a gradually increasing period (111 sec, as shown in Figure 4B). We have proposed that resetting from a slow to a fast rhythm reflects changes in the activity of a slow metabolic oscillator, which underlies islet oscillations in the “Dual Oscillator Model” [34], [36].

We also conducted similar resetting studies, but instead of treating islets with 0 mM glucose to shut down oscillations, we used 11 mM glucose containing 250 µmol diazoxide, a K_ATP_-channel opener that blocks [Ca^2+^]_i_ influx downstream of glucose metabolism. Interestingly, the application of diazoxide (DZ) was unable to replicate the action of glucose free even though DZ also transiently reduced [Ca^2+^]_i_ (Figure 4C). The islets in this case returned to their original [Ca^2+^]_i_ oscillatory period upon washing out diazoxide (Figure 4C). This difference in response is summarized in Figure 4D, and demonstrates that the resetting we observed upon removing glucose was likely metabolic in nature and not due simply to islet repolarization. Note that a decrease in amplitude in fura-2 ratio signal was observed throughout the recordings, which likely reflects a combination of dye loss, photobleaching, and/or photochemical production of non-calcium sensitive fura-2 species, as discussed previously [37], [38].

**Figure 6:**
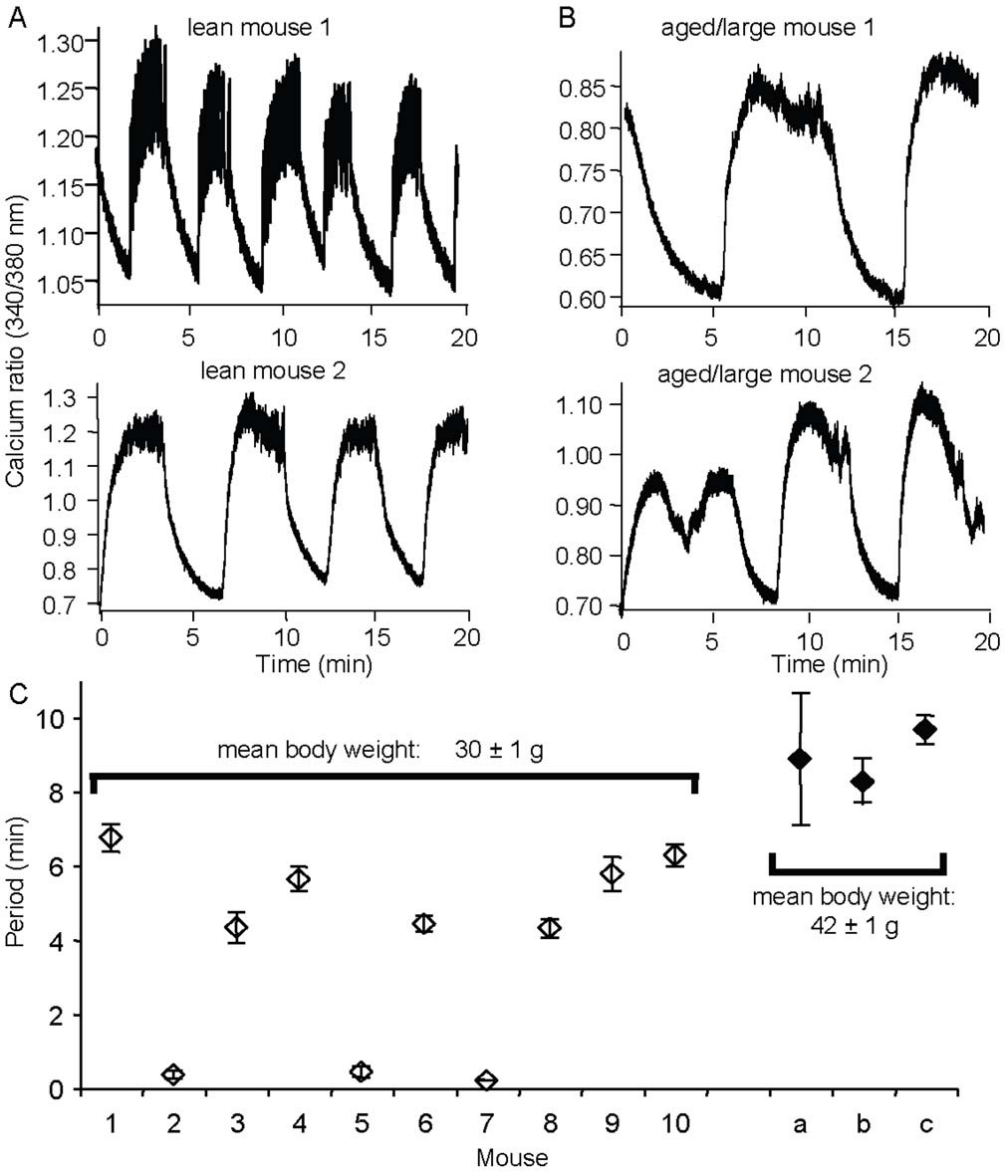
Effects of weight gain with age on imprinting. (A–B) Representative examples of islet [Ca^2+^]_i_ patterns in 11 mM glucose among lean mice weighing <30 g (A) and aged/large mice weighing >40 g (B). (C) Mean period of oscillations among 10 lean and 3 aged/large Swiss-Webster mice. Mean weight and mean period of islet [Ca^2+^]_i_ oscillations differed between groups (p<0.001).

### Oscillations in NAD(P)H Levels Provide Evidence for Metabolic Imprinting

We have suggested here and previously [20] that the slow oscillations are mediated by metabolic oscillations. We, therefore, tested whether imprinting could be observed in metabolic oscillations in addition to [Ca^2+^]_i_ oscillations. NADH is produced as glucose is oxidized in the reactions of glycolysis and the citric acid cycle, and can be detected (along with small amounts of NADPH, an anabolic reducing agent) in response to ultraviolet stimulation; thus it is more correct to say we have recorded NAD(P)H. We measured NAD(P)H in islets at 5 s-intervals from several mice as a marker of metabolism. Representative examples of oscillations in NAD(P)H from two different mice, Mouse 4 and Mouse 6 (Figure 5A), demonstrate two distinct rhythms. Fast Fourier Transform (FFT) was performed to determine the dominant oscillatory period for each islet measured, as demonstrated in Figure 5B for the two examples in Figure 5A. The dominant peaks on the FFT-generated power spectral density estimates correspond to NAD(P)H oscillations of ∼5 min for Mouse 4 and ∼9 min for Mouse 6. Among the 90 islets measured (Figure 5C), a substantial difference was observed in the mean period of NAD(P)H oscillations between two of the mice and the other four (P<0.001 by one-way ANOVA). These data provide clear evidence that NAD(P)H oscillations in islets differ from one mouse to the next, as we have observed in other intracellular processes.

### Imprinting and the Consequences of Age and Weight Gain in Mice

Metabolic changes are known to occur with aging and weight gain. We investigated the impact of these changes on islets by examining a set of Swiss-Webster mice that had grown to weigh 42.0±1.0 g (n = 3) as compared to control mice that only grew to a mean weight of 29.1±0.9 g (n = 10, p<0.001 for differences in body weight). Mice from both groups were ordered at the same time from the same vendor (Charles River Laboratory) and housed under identical conditions. As shown by the representative examples in Figure 6, the period of islet oscillations among the older mice (Figure 6B) was significantly longer than among islets from the same cohort of mice at a much younger age (Figure 6A). Mean values among all mice are shown in Figure 6C (p<0.001). The period increase was expressed across islets from a given mouse, indicating that imprinting continues even as the mice age and the islet properties change.

### Imprinted [Ca^2+^]_i_ Oscillations Are Difficult to Discern in Dispersed Beta Cells

**Figure 7:**
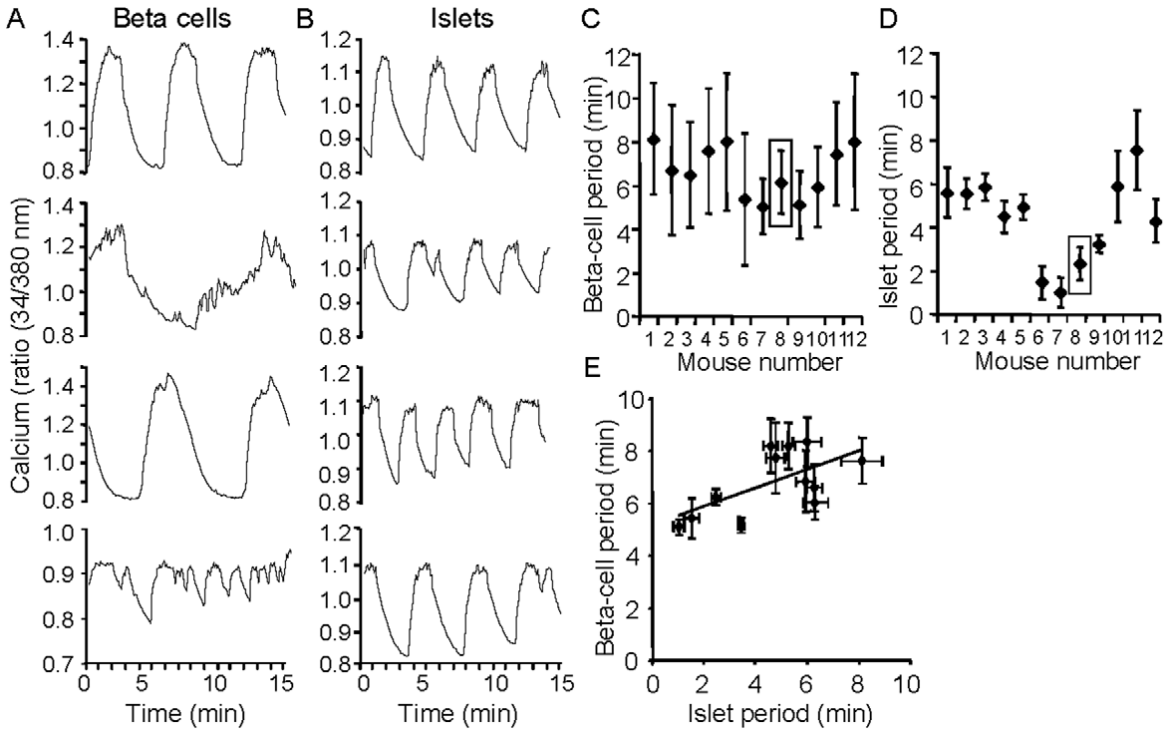
Dispersed beta cells do not display frank imprinting. (A) Representative examples of oscillatory patterns from 3 individual beta cells (A) and 3 islets (B) taken from the same mouse (Mouse 8 as indicated by the box in C). (C–D) Mean period ± SEM from 12 sets of beta cells (C) and corresponding islets (D) from the same mouse. Beta cells displayed longer period and also a greater degree of variability in their periods as noted by the large standard deviations they exhibited compared to islets. A total of 137 beta cells and 109 islets were recorded among 12 mice. One-way ANOVA indicates differences among beta-cell periods (P<0.01) and substantial differences among islet periods (p<1.0e-25) from mouse to mouse. (E) Scatter plot showing the relationship between oscillatory periods of beta cells and islets among the 12 mice studied (R^2^ = 0.39, p = 0.22).

We next examined whether imprinting was maintained among individual beta cells cultured from dispersed islets. Several islets originating from the same mouse pancreas were dispersed into individual beta cells and then cultured overnight on gelatin-coated glass cover slips. Intact islets from the same mouse were used for comparison of [Ca^2+^]_i_ oscillations. As shown in the representative examples in Figure 7A, single beta cells from an individual mouse displayed a wider variety of oscillatory patterns and wider range of oscillating periods when compared with islets from the same mouse, which showed much more uniform rhythms indicative of imprinting (Figure 7B). As summarized in Figure 7C, a comparison of the average oscillation period of dispersed beta cells for 12 separate mice showed minimal imprinting, as judged by the large error bars and homogeneous periods (ranging from ∼6–8 min). In contrast, a greater range of oscillatory periods (<1 min to ∼8 min) and smaller error bars were observed for islets from the same 12 mice (Figure 7D).

As observed by comparing Figure 7C and 7D, islets with the shortest periods (Mice #6–8) corresponded to some of the shortest periods observed among beta cells, suggesting that beta cells might retain a residual imprint. We next tested if a linear correlation existed between the period of islets and the period of dispersed beta cells among the 12 mice, but as shown in Figure 7E, the correlation was not significant (R^2^ = 0.39, p = 0.22). Additional statistical analysis by one-way ANOVA showed robust differences in the period of islet oscillations among different mice (p<1.0e–24), and Tukey post-test analysis of pairings indicated that 36 of 66 pairings differed in mean period among the 12 mice examined (p<0.05). Beta-cells also differed in period among these mice (p<0.01), but a post-test Tukey analysis revealed only 1 of 66 pairings differed significantly in mean period (Mouse 5 vs. Mouse 9, p<0.05). Thus, in contrast to intact islets, which clearly maintain imprinting, at best a trend toward imprinting can be discerned in isolated cells. Nonetheless, it is possible that the single cells are imprinted but that it is obscured by the increased variability of single cells compared to islets.

### Characterization of Pulsatile Insulin Release Patterns from Individual Islets

**Figure 8:**
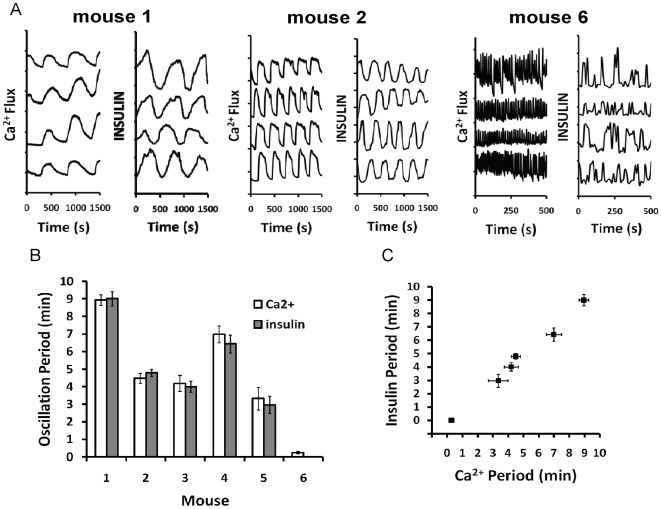
[Ca^2+^]_i_ flux and insulin release patterns show mouse-to-mouse imprinting. (A) [Ca^2+^]_i_ flux and insulin release traces from islets taken from three different mice (labeled accordingly). Displayed oscillation frequency averages are 9 min (Mouse 1), 4.5 min (Mouse 2), and 15 s (Mouse 6). Periods were calculated using local minimum values. Insulin oscillations from mouse 6 were faster than the measured temporal resolution (22 s) of the chip, causing under sampling of secretion dynamics. (B) Comparison of average [Ca^2+^]_i_ and insulin oscillation periods from each animal. Data sets are n≥6 islets and error bars are ±1 standard deviation. (C) Plot of average [Ca^2+^]_i_ versus insulin for each mouse. The linear relationship of data points suggests good agreement of oscillation frequencies (R^2^ = 0.98; p<0.0001).

Although oscillations in insulin secretion are known to be strongly correlated with calcium flux *in vitro* [20], [32], [39], to date, the physiological consequences of imprinted [Ca^2+^]_i_ patterns for insulin secretion have not been studied. This is primarily because the periods of the oscillations we observed were often faster than can be easily resolved using traditional islet perifusion methods. To overcome this limitation, we employed a recently developed microfluidic chip for characterizing insulin secretion dynamics in real time from multiple individual islets [26]. We tested at least 6 islets from 6 individual mice to assay [Ca^2+^]_i_ along with insulin release during continual superfusion with 11.1 mM glucose. Oscillations from three mice used in the study are shown in Figure 8A, which illustrates that similar frequencies were observed for islets taken from individual mice. Note that Mouse 6 displayed very fast [Ca^2+^]_i_ oscillations, but the corresponding insulin pattern was irregular and without detectable oscillations. It is possible that regular secretory oscillations occurred and were correlated to the fast [Ca^2+^]_i_ oscillations, but that the temporal resolution of the chip, estimated at 22 s, was too slow to detect them. Results were summarized by plotting the average of observed [Ca^2+^]_i_ oscillations and insulin release periods for each mouse (Figure 8B and C). These findings show two interesting trends: i) islets from single mice showed a strong correlation between [Ca^2+^]_i_ and insulin secretion patterns (R^2^ = 0.98; p<0.0001) and, importantly, ii) the oscillation periods were noticeably different from mouse to mouse. Note that Mouse 6 was included only on the graph for illustration using 0 min for the period of insulin oscillations; this data point was not used in calculations of R^2^ or p-value. These results agree with our previous study of *in vitro* [Ca^2+^]_i_ oscillations, and confirm that the pattern of insulin release from isolated islets is subject to the same imprinting as [Ca^2+^]_i_.

## Discussion

We previously reported that islets from a population of mice display a bimodal distribution of periods, with peaks in the fast (<60 sec) range and slow (>2 minute) range, but that islets from a given mouse have tightly clustered periods that fall into just one of those ranges. We interpreted this finding to mean that islets in a mouse are imprinted by some factor or factors that harmonize their periods. In this study, we explored whether islet imprinting is restricted to the outbred Swiss-Webster mice used in the first study. We also investigated whether islets maintain imprinted patterns of insulin release, as they do for [Ca^2+^]_i,_ using a novel microfluidics system which allowed insulin secretion to be measured from single islets with high temporal resolution. We showed further that imprinting is expressed in metabolic (NAD(P)H) oscillations as well as [Ca^2+^]_i,_ oscillations. Finally, we asked whether intact islet structure is important for maintaining the tight distribution of oscillatory periods among islets from the same mouse that is characteristic of imprinting.

### Imprinting Is Not Genetic in Origin

The experimental results presented here confirm our original report [20] and further suggest that islet imprinting is not restricted to outbred Swiss-Webster mice, as it can be observed in islets from outbred CD1 mice, as well as inbred C57Bl/6J mice. Examination of the periods of the islet oscillations for each of these strains confirms that slow and fast patterns are conserved among all the strains, as is bi-modality, though the proportions of fast and slow islets may differ. The finding of imprinting in the inbred C57/B6 strain indicates that the differences between fast and slow mice we observed are not genetic in origin, but it remains possible that they reflect variations in gene expression. This is further supported by the resetting experiments, which show that oscillation period can be dramatically reduced by removing and re-adding glucose in a brief experiment. We suggest that islets *in vivo* may be imprinted by the pattern of glucose or other regulatory factors to which they are exposed; that is, imprinting may reflect the metabolic history of an individual mouse. Such changes could result from changes in gene expression levels that bias the multi-potent oscillatory system towards either fast or slow oscillations.

### Imprinting of Insulin Secretion

Using a novel microfluidics system which allowed insulin secretion to be measured from single islets with high sensitivity and high temporal resolution, we found that islets maintained imprinted patterns of insulin release just as they do for [Ca^2+^]_i_. Thus, islets displaying faster [Ca^2+^]_i_ oscillations in turn had faster insulin oscillations, while slower [Ca^2+^]_i_ oscillations resulted in slower insulin oscillations. Insulin period and [Ca^2+^]_i_ period both measured *in vitro* were highly correlated, similar to our comparison of *in vivo* insulin pulsatility and *in vitro* [Ca^2+^]_i_ oscillations in our previous paper [20]. This suggests that imprinting extends to the patterning of insulin exocytosis from beta cells.

### Possible Physiological Significance of Imprinting *In Vivo*

Different insulin pulse patterning may differentially affect the metabolic homeostasis of the animals if the frequency of the insulin oscillations in turn determines how effectively secreted insulin suppresses hepatic glucose production, one of the major targets of insulin action. Long term, more prolonged insulin pulses may increase the risk of hepatic steatosis due to prolonged insulin action, as has been suggested [40], [41]. Differences in pulsatility could also affect the uptake of glucose by muscle and fat tissue [42]–[45].

While the extant data reported in the literature are variable, we hypothesize that changes in the dynamics of islet insulin pulsatility, in addition to changes in islet mass might also compensate for increased insulin resistance under homeostatic conditions [46], [47]. If this hypothesis is correct, then variable imprinted Ca patterns and concomitant insulin pulsatility in healthy mice may reflect attempts by the islet to compensate for varying levels of endogenous insulin resistance. It is thus tempting to speculate that different mice could be differentially susceptible to the development of diabetes or obesity based on their imprinted oscillatory patterns. Whether islet imprinting reflects factors such as peripheral insulin resistance, differences in body fat or even differences in food intake or metabolism remain to be determined. We did observe that older, overweight mice had slower rhythms than younger, lean mice but remained imprinted.

### Islet Architecture and Imprinting of the Oscillatory Patterns

Our data could not clearly establish that imprinting occurs at the level of the individual beta cell, even though isolated beta cells exposed to glucose display [Ca^2+^]_i_ oscillations. The [Ca^2+^]_i_ patterns we found for isolated beta cells in the present study showed wide variability and at best only a weak reflection of the imprinting process, even when the beta cells were isolated from mice whose islet oscillations had tightly distributed periods.

One would nonetheless expect the oscillatory period of a given islet to be determined by the average period of oscillation of its constituent beta cells. That is, a ‘fast’ islet, one that oscillates with a period of less than a minute, would be made up of mainly ‘fast’ beta cells, while a ‘slow’ islet would, in contrast, contain mainly ‘slow’ cells, those with periods >2 minutes or more. The Dual Oscillator Model, a mathematical model that can account for both the fast and slow oscillations and transitions between them [34], [36], suggests that in the fast cells an ionic oscillator is predominant, whereas in slow cells a metabolic, possibly glycolytic, oscillator predominates. The model proposes that the slow oscillations are mediated by positive feedback of fructose 1,6 bisphosphate onto the muscle isoform of the allosteric glycolytic enzyme PFK. Thus, slow cells may express this isoform of PFK to a greater degree. Alternatively, differences in the activity of glucokinase (GK) or other metabolic proteins could also account for the differences in period. The model further suggests that if the population of mice varies broadly in the proportions of fast and slow cells, a bimodal distribution of their oscillation periods would emerge naturally (cf. analogous results with a different model, Fig. 2 of [48]). If, on the other hand, the proportion of fast and slow cells in the islets of a given mouse is tightly controlled, i.e. the cells are imprinted, then the islets from that mouse would display similar periods, as we observe.

We have interpreted our data assuming that the fast, slow, and mixed patterns represent the intrinsic properties of individual beta cells. For an alternative view that stresses paracrine interactions between beta and alpha cells rather than the synchronized activity of beta cells, see [49], and also [50]–[52].

As slower islets showed a trend, albeit non-significant, to have slower single cells than faster islets, it is possible that the oscillation periods of single cells do reflect imprinting, but that this is obscured by the vastly increased heterogeneity of the single cells apparent in Fig. 4 and anticipated from theoretical modeling [53]. We also lack a credible way to prove that the differences between the oscillations of single beta cells and islets were only due to a lack of coupling, rather than loss of some necessary factor in the islet in the process of cell isolation. In fact, we consistently found many more isolated cells having slow periods compared to the islets from which they came. Thus, it may be that intact islet architecture is necessary for a tight distribution of periods and not just the averaging of heterogeneous cell properties.

### Conclusions

Our findings demonstrate that the islet imprint phenomenon is a robust and general feature of islet function in rodents that warrants further investigation as a possible indicator or even an instigator of beta-cell dysfunction. While the imprinted patterns are unlikely to be explicitly genetic in origin, they may indirectly reflect changes in the patterns of gene expression of individual mice that in turn bias the system towards the production of either fast or slow oscillations in response to changes in the metabolic history of the animal. Thus, these findings may be directly relevant to diabetes, obesity and other metabolic disorders.
